# Medicine, emotience, and reason

**DOI:** 10.1186/s13010-024-00154-y

**Published:** 2024-04-10

**Authors:** John F. Clark

**Affiliations:** 1 UCSF, Natividad Medical Center Family Medicine Residency Program, 1441 Constitution Blvd., Salinas, CA 93906 USA; 2 UCSF Medical School, 533 Parnassus Ave., San Francisco, CA 94143 USA

**Keywords:** Emotion, Intelligence, Knowledge, Reason, Diagnosis, Dual-process, Compassion, Meaning, Burnout, Dehumanization

## Abstract

Medicine is faced with a number of intractable modern challenges that can be understood in terms of hyper-intellectualization; a compassion crisis, burnout, dehumanization, and lost meaning. These challenges have roots in medical philosophy and indeed general Western philosophy by way of the historic exclusion of human emotion from human reason. The resolution of these medical challenges first requires a novel philosophic schema of human knowledge and reason that incorporates the balanced interaction of human intellect and human emotion. This schema of necessity requires a novel extension of dual-process theory into epistemology in terms of both intellect and emotion each generating a distinct natural kind of knowledge independent of the other as well as how these two forms of mental process together construct human reason. Such a novel philosophic schema is here proposed. This scheme is then applied to the practice of medicine with examples of practical applications with the goal of reformulating medical practice in a more knowledgable, balanced, and healthy way. This schema’s expanded epistemology becomes the philosophic foundation for more fully incorporating the humanities in medicine.

*Illness brings ardor*

*Rationality reserve*

*Passion compassion*

## Introduction

Medicine is founded on emotion. To be affected by those who suffer and moved by the urge to care is the *raison d’être*of the profession. Empathy, to feel within for another, is among the most highly valued mental acumen in medical practitioners. There is considerable evidence that compassionate care is more efficient and satisfying [1]. Yet, a formal discourse in professional emotions barely exists much less instruction in passionate medical engagement. Indeed, the hidden curriculum of medicine generally instructs strident emotional suppression [2]. Instead, formal medical education and practice focus nearly exclusively on cool rational thought regarding objective scientific knowledge applied in unbiased intellectual analyses of patients and their illnesses.

Such a strong preference for dispassion in medicine inherently marginalizes passion and compassion in the profession and expresses a deep philosophic bias toward intellect and against emotion with regard to medical reason and practice. It will here be argued that this bias is problematic. Medicine is diminished and made unwell by hyper-intellectualization; There is now a “compassion crisis” in medicine [3, 4]. Through the labor of emotional distancing, many a modern medical mind has become afflicted with an exhausting occupational mental illness [5]. The marginalizing of humanity’s humane feelings for each other has led to a dehumanization of medicine [6]. By way of its dispassion, medicine is struggling with meaning [7]. Such professional problems demand deep reflection on medical training and practice that have profound philosophic dimensions. These dimensions extend well beyond medicine to general questions regarding human knowledge and reason. Modern solutions will need to be both medically and philosophically novel.

Medicine’s current bias toward intellect and against emotion in medical reasoning and practice is here considered not only dysfunctional but philosophically unwarranted. In response to this assessment, this work will briefly outline a novel philosophic schema for incorporating human emotion equally alongside human intellect in human knowledge and reason. This schema will then be applied to the practice of medicine with an eye toward reformulating medical practice for the better and addressing its current hyper-intellectualized problems. This undertaking will afford medicine the opportunity to not only better itself but also provide leadership in enabling Western culture to become more fully compassionate. In the service of such a humane social effort, this work will briefly consider hyper-intellectualization in Western culture before moving on to that of medicine.

## Hyper-intellectualization in philosophy

To be fair, medicine isn’t entirely to blame for its disaffection. There are social determinants. Modern Western medicine is embedded in a modern Western post-enlightenment culture that’s become increasingly hyper-intellectualized [8]. For medicine to regard the limitations of its dispassion and reengage professional emotions in a healthy way, it will not only need to consider hyper-intellectualization in its own philosophy but contend with hyper-intellectualization in the broader culture.

Indeed, the philosophic roots of Western hyper-intellectualization are ancient and deep. Some highlights; Democritus, the pre-Socratic philosopher considered by many to be the father of modern science for his atomist theories of matter, called knowledge generated through sensual experience “bastard” knowledge while that through intellectual thought “legitimate” knowledge [9]. Zeno, the founder of stoicism, elevated the unaffected mind in terms of the classic stoic calm thus, “A bad feeling is a commotion of mind repugnant to reason.” [10] Descartes’ struggle with knowing led to his seminal, “*Cogito ergo sum*—I think therefore I am,” [11] a thought that conspicuously excludes feel from the realm of self-evident truth. Spinoza may have been the most strident in declaring the primacy of intellect in his Ethics,

Without intelligence there is not rational life: and things are only good, in so far as they aid man in his enjoyment of the intellectual life, which is defined by intelligence. Contrariwise, whatsoever things hinder man's perfecting of his reason, and capability to enjoy the rational life, are alone called evil [12].

Modern philosophy has generally continued in this hyper-intellectualized vein. Nietzsche's nihilism dismisses morality by employing the standard Western dismissal of emotion, stating, “(M)oralities are … merely a sign language of the affects.” [13]. Wittgenstein’s Tractatus, which heralded the broad arc of 20th-century philosophy, has been characterized as a linguistic turn toward analytic philosophy [14], an inherently hyper-intellectualized formulation. Emotionality has been modernly formulated as a kind of intelligence rather than as a mental acumen unto itself [15]. De Sousa, in his The Rationality of Emotion, excludes emotion from cognition by simply stating “emotions are not beliefs” [16] and thus can’t be justified and true. Even modern dual-process theory, which purports to incorporate emotionality and intuition into mental assessments, subsumes them under an intellectual paradigm, exemplified by Kahneman’s book Thinking Fast and Slow [17]. Yet the intuitive/emotional mind doesn’t think; it feels. Further, Kahneman’s automatic/emotional/intuitive “system 1” is often assigned pejorative descriptors such as primitive and sloppy while his controlled/rational/logical “system 2” is considered more advanced and accurate.

## Hyper-intellectualization in medicine

Regarding medicine, modern medical hyper-intellectualization is ubiquitous. While emotions are officially acknowledged and valued in medicine largely in the form of professional empathy, they are generally handled superficially and abstractly if not dismissively. Consider the following.

At the outset, medicine attracts aspirants who are not only intelligent but caring, compassionate, and affected by the suffering of others [18]. It then thrusts these naturally sensitive individuals into the pitched theater of human illness with its unavoidably intense emotion and overwhelming affect. It goes on to demand trainees don the mantle of cool rationality and be dispassionate in their intellectual analysis of patients and unaffected in their application of medical treatment [19, 20]. As if all the repression needed for that intellectualization weren’t problematic enough, the profession then provides its sensitive practitioners little in the way of formal outlets to express medical passions and, worse, offers them a hidden curriculum that generally instructs strident emotional suppression [2, 21, 22]. Finally, medicine now sends its members out into work environments that are increasingly industrialized and technologically immersed—monetarily efficient health conveyors that employ a multitude of unbiased population-based metrics serving myriad institutional agendas. These hyper-rational transactional environments treat physicians as interchangeable parts in standardized health mills and expect them to fill generic isolated roles that are, by design, insensitive to any individual practitioner’s unique tender emotional needs in managing the affective load of medical practice. Medicine’s passions and compassions have become collateral damage in this process.

Current professional reflections on medical reasoning and diagnosis are nearly exclusively framed in terms of intellectual processes such as thought, thinking, analysis, logic, and rationality [23]. Marginalized is the idea of incorporating clinical intuition into diagnosis with its feel and sense of things much less any contribution of emotions themselves to a thorough evaluation of patients and their circumstances [24]. Medical knowledge is currently framed largely in terms of evidence-based medicine, with evidence in this context referring to rational conclusions drawn from objective, quantitative, population-based scientific studies. In this paradigm, neither the clinician's own subjective thoughts nor relational feelings are considered evidential to medical knowing. Current formal medical epistemologies, while varied, tend to emphasize traditional linguistic-based propositional knowledge and intellectually justified true beliefs where justification is a function of examined controlled logical thought rather than unexamined spontaneous intuitive sense [25]. Further, medical judgments have become progressively more influenced by standardized population-based guidelines handed down from abstracted authoritative bodies [26]. This dynamic inherently marginalizes the conscience-based ethical feelings arising from the practitioner’s own immediate body during the course of the relational care of unique patients [27].

But is any of this dysfunctional? One need not look far to find troubling consequences of the current hyper-intellectualization in medicine. Some examples; In light of the rationally formulated traditional aims of medicine or the more recently formulated (managerial) quadruple aim [28, 29], inefficiency abounds. The number needed to treat (NNT) for many if not most standard medical therapies is easily into the tens if not hundreds [30]. Medical expenditures on modern medical technology sired by scientific inquiry have grown unsupportable and had diminishing health benefits [31, 32]. There is a crisis of medical meaning. One hears of efforts to “find meaning in medicine” [6]. Where was it lost? Many medical professionals are suffering from an exhausting occupational mental illness that is worsening [33]. While not currently formulated as emotional in etiology, burnout is rife with symptomatology evincing mental dysfunction born of excess dispassion; depersonalization, detachment, loss of empathy, emotional exhaustion, emotional distancing, and cynicism.

Such hyper-intellectualized professional dysfunction entails profound questions for the philosophy of medicine if not Western philosophy in general regarding no less than the human mind and what constitutes human reason. Not the least of such questions would be; “What is intellect?” “What is emotion?” and “What is reason?” In the service of advancing the profession, here is proposed the beginning of an answer born out of the crucible of medical practice.

## A novel schema of human reason

It’s presented in the form of a briefly summarized novel schema of human knowledge and reason that demands an equal balance between emotion and intellect to be knowledgeable and reasonable. The schema is novel and thus unreferenced. Historically, definitions of human intellect, emotion, and cognition have been varied and conflicted. Remarkably, no broad consensus exists. Indeed, in regard to emotion themselves, neuroscientist Joseph LeDoux observed,

“Unfortunately, one of the most significant things ever said about emotion may be that everyone knows what it is until they are asked to define it.” [34]

This work considers most current schema of human cognition inadequate by way of hyper-intellectualization with its inherent marginalization of emotion. The following philosophic schema will generally address Western hyper-intellectualization by extending dual-process theory [35] to not only provide a definition of intellect and emotion in terms of their distinct natural process and function but to define each as independently epistemic. The schema extends current cognitive theories of emotion [36] and affective epistemology theories [37] to consider emotions as not only supporting general human cognition and knowledge but as being independently cognitive and epistemic in their own right apart from intellect. The schema also extends dual-process theories by more fully considering the conflicted interaction between these two distinct kinds of cognition. This fills a gap in current epistemology regarding emotional knowledge and permits a broader less hyper-intellectualized and more mentally balanced discourse in human knowledge and its practical application.

The schema is novel in that it employs a “the means condition the ends” argument to propose not only two distinct natural kinds of process in the human mind, here termed intelligence and emotience, but two distinct natural kinds of knowledge, intellectual and emotional. The schema details the distinct characteristics of these two natural kinds of process and knowledge. It also observes the distinct phenomena through which each is metacognitively experienced as well as the distinct medium through which each is socially communicated.

The schema goes on to outline how the emergent interaction of human emotion and intellect together constructs general human cognition and reason. This is not a blended interaction but rather a yin-yang-like dynamic between thoughts and feelings. In this ever-unfolding dynamic, feelings and thoughts, while independent, are in constant interaction with each other; feelings lead to thoughts that stimulate more thoughts which then go on to trigger further feelings that themselves stimulate more feelings and thoughts, etc. In this schema, the terms “intuitive thinking” and “rational sense” are incoherent. Rather intuitive sense and rational thought, while independent, work in close relation to form something greater than their sum.

In terms of competence, a distinct competence is necessary to interact with each of intellect and emotion. A further competence is needed in how they interact together to form not just emotional intelligence but intellectual emotience and ultimately emergent human reason. The goal of this undertaking is to provide an adequate schema of human knowledge and reason that is more complete, healthy, and functional than current ones.

The following philosophic schema will then be applied to medicine to address the issue of medical hyper-intellectualization with an eye toward providing an adequate philosophy for clinical practice. The following schema is meant to reformulate medicine by providing a philosophic foundation upon which to build a more thoroughly knowledgable and mentally healthy practice through better integrating emotions and intellect professionally.

First intellect. The function of human intellect is to solve problems. Human intelligence employs a willfully driven mental process assessing perceptual input that is by nature conscious, controlled, slow, abstract, and reductionist (analytic). The intellectual mind is designed to produce rationally justified true beliefs that represent objective, timeless, mechanistic knowledge (of how things work) that enables one to eventually exert control over the future (i.e. plan). This process is generally referred to as thinking. Technology is its byproduct. Intellectual beliefs are thought beliefs and phenomenally experienced as a thought. They are expressed/communicated linguistically with the tongue (Latin *lingua*) in terms of reductionist language using spoken/written literal words. Intellectual knowledge is thus inherently logical (from Greek *logos* “word”) and recorded using prosaic written words. Such a mental process of necessity considers invalid its converse.

Emotion. The function of human emotion is to be moved by value. Human *emotience* employs an instinctually driven mental process assessing perceptual input that is by nature subconscious, spontaneous, fast, relative, and holistic (synoptic). The emotional mind is designed to produce intuitively justified true beliefs that represent relational, immediate, precious knowledge (of value, from Latin *precium* “value, price, worth”) that enables one to instantly react beneficially in one’s current circumstance. This process is generally referred to as feeling. Reflex action is its byproduct. Emotional beliefs are felt beliefs and phenomenally experienced as a sense. They are expressed/communicated corporistically with the entire body (Latin *corporis*) in terms of holistic corporage using nonverbal symbolic communication. Emotional knowledge is thus inherently symbolical and recorded most purely using sensual representational aesthetics (from Greek *aísthēsis* “feeling”). Additionally, emotional beliefs are also recorded less purely using language in terms of figurative poetic and circumstantial narrative.

Over both of these informationally encapsulated assessment modules (Griffiths) [38] reflecting the functional architecture of the human mind (Pylyshyn) [39] resides the global prudential self. This global self employs the overarching, emergent, human mental capacity to self-perceive intellectual and emotional processing and use the products of both to volitionally guide choices regarding internal process and external action. This global metacognitive capacity is none other than human reason. Reason is not equated with rationality. This is a constrained and intellectually biased idea. Rather, human reason is the dynamic interaction between intellect and emotion working together. Each provides what the other cannot and both together expand human mental capacity beyond either alone. Further, human reason does not arise from merely a part of the mind/body (i.e. brain). This is a reductionist formulation. Rather, human reason is the whole of the human body/mind working in tandem through a unified dynamic between feelings and thoughts together [40]. This overarching, holistic, emergent, reasonable human capacity is what enables humanity to be prudential and make wise choices. It's what permits each of us to be a philosopher (Greek “lover of wisdom”) and all of us together to be *Homo sapiens* (Latin “wise earth dwellers”).

The fact that intellect and emotion employ complementary and mutually exclusive ways of processing perceptual input has consequences. First, it forces the global self to consciously attend to either one or the other at any given moment, to either analyze or evaluate. Second, because intellect and emotion represent different natural kinds of mental processing, each produces different natural kinds of knowledge. In other words, each independently provides knowledge that the other cannot. In particular emotions, apart from any thought whatsoever, express their own natural kind of knowledge through feelings. To use one to try to know the knowledge of the other is like trying to use a telescope to smell a flower. It's simply impossible. This is not to dismiss intellectual assessments with their inherently objective, reductionist, mechanistic beliefs and knowledge, just to consider them alone inadequate for a thorough knowing of existence. Third, each kind of knowledge is invalid within the frame of the other, and to bring them together there needs to be a translation of each kind of knowledge into the other’s frame (e.g. thought < — > feeling, linguistic < — > aesthetic).

The ability to prudentially guide beneficial action will, of necessity, depend on a distinct competence in each kind of knowing as well as how they both work together to make the self most knowledgeable and mentally capable in choosing action. This may involve temperance over emotionally urged behavior. It may also involve letting go of intellectually generated thoughts and plans. Indeed, depending on one’s circumstance, the prudence of the global self may just as reasonably choose to give oneself over to immediate instinctually urged action as to intentionally execute planned willful action.

An additional consequence of the complementarity of intellect and emotion is that they conflict. The global prudential self must manage this inherent mental conflict internally in order to be reasonable. Willful control is central to the conflict. The conflict is not only an internal one between controlled rationality and spontaneous intuition but an external one between one’s behavior being under conscious willful control or subconscious instinctual control. This conflict is at the front lines of the existential conflict between what is humanly controllable and uncontrollable in existence. Intellect provides willful knowledge of what can be controlled; or effect. Emotion provides spontaneous knowledge of how one is controlled; or affect.

This conflict between being effective and affected is perhaps the most fundamental conflict of any living organism; the conflict between controlling one's fate or submitting to it. It is a conflict that casts the human mind internally at the tense liminal threshold between the supra-liminal mind and sub-liminal mind; between cool rationality and warm passions, between knowing intentionally and knowing spontaneously, between intellectual hegemony and the wilds of the mind. Externally, this existential conflict casts the individual at the border between one’s own will and the will of things beyond it; between one’s chosen order and the order of things given, between what one can create and how things have been created. Behaviorally, the conflict casts the self at the frontier between choosing controlled action or letting go to one’s passions; between disciplined restraint or spontaneous abandon.

The Western philosophic bias toward intellect in this conflict can thus be seen as a bias toward willful control over one’s own mental processes, one’s own actions, and further one’s destiny. This controlling bias is what’s led Western culture to become adversarial to the spontaneous, the uncontrolled, and the wild. This bias for control is what’s led Western philosophy to become estranged from spontaneous human emotions—from our passions and compassions. The controlling bias is also what has constrained dual-process considerations of cognition to only thinking. Given the preponderance of Western philosophic work (linguistic-based) on intellectual knowledge and reasoning, achieving balance and peace in this philosophic conflict of mind will generally involve 21st-century Western philosophy making a corporistic turn toward synoptic philosophy. This will require developing a better understanding of emotional knowledge and refining human reasoning through a more robust discourse in aesthetics, poetics, and narrative. Western culture will need to create a more humane formulation of how emotions and intellect work together in peace to make us most reasonable, wise, and healthy [41].

## A balanced schema of medical reason

Applying this schema to medicine now affords the opportunity for the profession to reformulate itself for the better by balancing the medical mind through more fully incorporating human emotions into medical practice. This reformulation involves conceptualizing emotions as assets that provide knowledge independent of intellect and incorporating that emotional knowledge alongside intellectual knowledge into medical reasoning, diagnosis, treatment, and care. Currently, such a conceptualization of emotions as being independently epistemic is absent from medical philosophy and thus medical practice.

Medicine is uniquely situated to find peace in the conflict between intellect and emotion. Given medicine’s compassionate role in solving medical problems and exerting control over natural illness, medicine naturally exists at the ardent conflicted frontier between what is humanly controllable and uncontrollable. Medicine’s bias toward intellect is a bias for control over the wilds of natural illness and death. This is certainly understandable given medicine’s traditional role. Yet it is here considered self-evident that humanity is neither fully in control of nature nor of illness and death. The uncontrolled remains a manifest natural human reality. Medicine knows this sober reality like no other profession.

Medicine is thus inherently conflicted. The ardent conflicted theater of human health and illness casts the medical mind externally at the conflicted frontier between human life and death and internally at the conflicted frontier between humanity’s controlling intellect and spontaneous emotions. This medical milieu is a maelstrom of both effect and affect. It is at this tense frontier that medicine needs to find a healthy reasonable balance of mind through developing a wisdom of peace in the conflict between the controlled and the uncontrolled in medicine and in human existence. Internally, this will of necessity involve a medical peace between humanity's domesticated controlling intellect and its wild uncontrolled emotions. Such medical wisdom will benefit both patients and practitioners. Once achieved, the profession can then offer such wisdom to the rest of the culture for the benefit of all. To do this, medicine will first need to engage the wilds of its own professional emotions and learn to reasonably incorporate them into civilized medical practice in a healthy way.

Medical philosophy needs found such efforts. Medicine’s current hyper-controlling mental bias is the genesis of its hyper-intellectualized dysfunction and the current compassion crisis in medicine. Yet human passions are the ground of human compassions. For medicine to grow beyond its current hyper-intellectually constrained epistemology and make itself more clinically knowledgable, whole, and healthy, medicine will need to move beyond its exclusive reliance on humanity’s controlling intellect to develop a parallel professional discourse that’s accepting of knowing through spontaneous human passions and compassions. This will require broadening medical reason beyond the strictly intellectual and into the emotional by expanding medical discourse beyond a strictly rational scientific discourse in human effect to equally include an intuitive humanistic discourse in human affect. Such a sober balanced professional discourse will enable consideration and acceptance of not only what is medically known and controllable but what is medically unknown and uncontrollable. It will permit a balanced discourse between medical knowing and mystery and develop a professional comfort with both ambiguity and unknowing in medicine [42].

Given medicine’s current hyper-intellectualization, achieving this balance will require considerably more professional philosophic work with dual-process in the medical mind and in particular with emotional knowledge in medicine and how to better integrate professional affect into medical philosophy and medical practice. This is not to say medicine needs to *be* more emotional. Indeed, medicine is already, by nature, a highly emotional milieu. This is humanly unavoidable. Rather, the question for the profession will be how to find novel ways to humanistically accept and integrate all the abundant clinical affect of medical practice into medicine in a productive, knowledgeable, and healthy way.

## Practical applications of emotience in medicine

What might that look like, and what would be its benefits? Central to such an effort would be to consider affective competence as necessary for clinical competence [43]. Physicians would need to strive to be not only intelligent in their work but *emotient* as well. Clinical practice would involve medical professionals having a healthy and reasonable interaction with not only patients externally but with themselves internally in the form of their relationship with their own mind.

With regard to being emotient, this would at least require medicine to regularly confess its own ever-present ardor and welcome it into ongoing professional discourse rather than ignoring it or relegating it only to the private sphere of pillow talk or weekend confidences over coffee. Professionally sanctioned practices would need to be developed that assist clinicians in regularly identifying clinical emotions and then embracing, sharing, and indeed utilizing professional passions in daily practice for the compassionate benefit of both patients and practitioners. The philosophy of medicine will need to show the way by assisting physicians in interpreting professional emotions in terms of the knowledge they provide and their contribution to clinical reasoning. Developing such wisdom would then create a path forward to integrate the knowledge contained within professional passions to make medicine better and, in particular, enable medicine to become more fully compassionate and healthy.

Empathy, that most valued affective medical acumen, could be explicitly supported and developed in practitioners, and the anti-empathetic elements of modern clinical environments limited. Nurtured would be a professional sensitivity to clinical emotion and an awareness of medical pathos [44]. Trainees could be taught to explicitly reflect back to patients empathetically registered relational content and utilize such emotion to facilitate a more compassionate medical encounter, as in “I can see you're frustrated by your dysarthria.” Beyond empathy, practitioners could be encouraged to explicitly share with patients not only their own analytic medical thoughts but also their own benevolent clinical feelings regarding the patient’s illness, as in “I’m very worried about your abdominal pain.” Such sharing with patients of how their suffering affects the practitioner would humanize the medical encounter and directly express medicine's inherent kindness. Such training would assist medical schools in a process of professional identity formation that explicitly incorporates empathy and passion in a mature professional practice.

The patient experience initiatives of Medicare and other insurers, which have already become fixtures of clinical practice and reimbursement, could be further refined to more directly capture the emotional/empathetic/compassionate meaning of the effort. Patient satisfaction surveys might explicitly ask if the practitioner was emotionally engaged and compassionate thus tying affective competence to professional competence and reimbursement. This would require medical systems to invest in compassionate care and redesign clinical environments, schedules, and reimbursement to accommodate professional compassionate connections as well as assist physicians in assimilating and utilizing the immense affective load of medical practice.

Rather than limiting medical reason to rational assessments that through a reductionist medical gaze formulate patients as broken biological machines to be fixed [45], the profession could also reasonably evaluate patients’ afflicted circumstances emotionally to holistically see how their illness affects their lives and engage with the values that move their choices [46]. There’s considerable evidence that such compassionate assessments are more medically efficient [1]. The close association of emotionality and intuition would need to be recognized, and clinical intuition would be explicitly developed. More work could be done to further develop dual-process models of medical knowledge and decision-making that better incorporate emotion [47]. Medical diagnosis (Greek *dia-* “through" + *gnosis “*knowledge”) would depend not only on rational thought but intuitive sense and bring together disciplined medical logic with instinctual medical feel in the dynamic formation of a thorough knowing of patients and their afflicted circumstances.

There would need to be developed and taught a deeper sophistication in translating clinical emotions into intellectual terms to facilitate intellectual understanding of affective content and thereby enable more balanced and reasonable clinical assessments and decisions. In particular, since emotions express beliefs about the gain and loss of things of value, emotions would ultimately need to be linguistically translated into those terms. For example, to a patient’s unwelcome cancer diagnosis, the instinctually horrified response from the patient might initially be expressed linguistically as, “I just want to run away.” This behavioral expression could be empathetically translated to “I’m afraid.” and then epistemically understood with the general propositional belief “I am threatened with the loss (of value)” and further understood in light of the patient’s own foundational beliefs regarding value in their experience, such as “My life has value.” This would enable a deeper evaluation of what in particular is valuable in a patient’s life in order to help the patient-doctor couplet refine and individualize difficult upcoming quality-of-life decisions [48]. Emotional engagement facilitates such ardent epistemic considerations of the manifest reality of value in patients’ and practitioners’ lives [49] and would eliminate the nihilism (denial of value [50]) inherent to unbalanced medical hyper-intellectualization. In this way, medical evidence and knowledge would become equally intellectual and emotional.

Rather than the objectified patient-centered formulation of medicine that isolates the patient and marginalizes the physician, promoting a relational patient-doctor-centered formulation would invite a regard for how each affects the other and how both cooperate to face illness together through shared decision-making. Medical rhetoric in the form of written H&Ps (History and Physical) and SOAP notes (Subjective, Objective, Assessment, Plan) could expand beyond their currently abstracted technical form to freely express relational content in the medical narrative, both the empathetically observed affect of patients and the felt humanistic emotion of practitioners, as in; “CC: The patient presented worried about epigastric pain,” “DI: The Head CT showed no intracranial abnormality. That was a relief,” “A: 1. Stroke—sadly the patient has a dense left hemiparesis,” or “P: It is hoped that aggressive IVF and IV piperacillin/tazobactam will resolve the patient's sepsis.” There could be developed EMR drop-down menus that contain affective lists of various emotions to choose from while documenting the clinical encounter. These might include; fearful, happy, sad, frustrated, resentful, relieved, hopeful, etc.

This kind of professional emotional expression would have to be professionally refined. Just as practitioners are generally trained to avoid intellectually unproductive and unprofessional terms such as “lying drug seeker” in the medical record, similarly medical professionals would need to learn to avoid unrefined affective terms such as “upset” or “pissed” in favor of more sophisticated emotional terms that express professional empathy and support. Such affective medical records would more thoroughly document the constructive professional passions inherent to the therapeutic relationship and memorialize medicine’s compassionate meaning. It would also formalize a professional sharing of clinical emotion within the medical community itself. This would help overcome the loneliness and isolation inherent to medicine's current hyper-intellectualized culture of affective neglect [51]. It would also promote a more collective professional support for compassionate care and connection.

Medical practitioners could reformulate themselves as not only excellent and virtuous in their work, but vulnerable. Burnout would be reformulated to become the Clinician Stress Disorder, a distant cousin to PTSD born in part of the mental effect of accumulated ongoing empathetic trauma endured through the constant close witnessing of patient suffering and loss coupled with the imposed labor of inhumane affective suppression [52]. In this formulation, a practitioner’s efforts to manage medicine’s affective load by becoming unnaturally hyper-intellectual within medicine’s naturally hyper-emotional environment would be recognized as mentally exhausting and indeed wounding. Compassion fatigue would here be analogous to battle fatigue. The emotional release of passionate professional expression would be recognized as necessary for professional health. There is good evidence that emotional expression after traumatic events is psychologically beneficial [53]. This is where shared passionate expression through the medical humanities has evidence of mental benefit and could become central to a healthy, compassionate, professional curriculum and culture [54, 55]. Indeed, emotionally expressive medical notes could become regarded as tools not only for patient health but for practitioner health as well! Such professionally refined emotional expression on the part of the physician in the medical record would not be self-gratifying but rather a professional aid to staying mentally healthy in medicine. It would also memorialize medicine's humanistic identity and embody in the medical record Pellegrino’s famous observation that medicine is “The Most Humane of Sciences, the Most Scientific of the Humanities” [56].

Such a reconceptualization of physician burnout is both needed and profound. Current formulations of burnout are having limited success, and there have been calls for better models to mitigate burnout in physicians [57]. The radically profound idea behind burnout is that the physician herself is made ill through medicine; the profession makes patients out of its practitioners. Samuel Shem’s “the patient is the one with the disease” is essentially refuted in this idea.; the physician is made the one with the disease through modern hyper-intellectualized medical practice [58]. While there can certainly be an unhealthy excess of empathy (as Shem’s statement implies), there can also be an unhealthy excess of dispassion with its inherent emotional neglect. A mindful balance is needed—a humanistic balance. Reconceptualizing burnout to include in its etiology imposed hyper-intellectualization, excess objectivity, and dysfunctional detachment is here proposed as a needed reformulation to improve the prevention and treatment of suffering imposed on medical professionals by a philosophically disordered medical system. Such a novel and profound reformulation necessitates an equally novel and profound reformulation of medical philosophy and indeed general human philosophy.

## Reformulating the aims of medicine

Ultimately, all this will mean reformulating the aims of medicine. In order for the profession to make itself more whole and healthy and sustain its physicians in terms of their passions for healing and compassions for patients, medicine will need to formally aim to be more explicitly emotional. This will mean expanding the aims of medicine to not only strive to be intellectually effective but allow for being emotionally affected. Fortunately, while a universal human mortality means extending life is ultimately unachievable in any patient, compassionate care is always achievable in every patient. Compassionate care also enables medical practitioners to care for each other and themselves. This is a hopeful reality.

Human passions hold deep within their affect a knowledge of value that can only be known emotionally. Similarly, human compassions hold deep within their pathos a knowledge of the value we each have to one another that can only be known emotionally. For the patient-doctor couplet to become more humanely productive, it will need to be more precious and less mechanistic; more humane and less transactional as Curin and Tollefsen point out in their The Way of Medicine [59]. For medicine to live up to its compassionate potential, the passionate knowledge of the natural value of human life must stand shoulder-to-shoulder with any intellectual knowledge of the natural workings of the human body.

It is in expressed human compassion that is found the beauty of medicine and the humane wisdom so needed by humanity to heal ourselves, our societies, and our world. For medicine to fully embody such wisdom, it will need to undergo a balanced rational/affective refoundation of itself through an expansion of medical philosophy and a more robust discourse in the medical humanities. Only then can the profession grow more thoroughly humane, whole, and well. Through its own passionate professional example, medicine can then go on to take leadership in encouraging society to become more fully compassionate, cooperative, and healthy.

## Data Availability

Not applicable.

## References

[1] Trzeciak S, et al. Compassionomics: The Revolutionary Scientific Evidence That Caring Makes a Difference. Pensacola: Studer Group; 2019.

[2] Hafferty F W, Franks R (1994). “The Hidden Curriculum, Ethics Teaching, and the Structure of Medical Education.”. Acad Med.

[3] Lown Beth A. (2011). “An Agenda for Improving Compassionate Care: A Survey Shows about Half of Patients Say Such Care is Missing.”. Health Aff (Millwood).

[4] Trzeciak, Stephen. “Healthcare’s Compassion Crisis.” Stephen Trzeciak: Healthcare’s Compassion Crisis / TED Talk, https://www.ted.com/talks/stephen_trzeciak_healthcare_s_compassion_crisis_jan_2018

[5] Shanafelt TD, Boone S, Tan L, Dyrbye LN, Sotile W, Satele D, West CP, Sloan J, Oreskovich MR (2012). Burnout and Satisfaction With Work-Life Balance Among US Physicians Relative to the General US Population. Arch Intern Med Archives of Internal Medicine.

[6] Haque Omar Sultan, Adam Waytz (2012). “Dehumanization in medicine.”. Perspect Psychol Sci.

[7] one example of many - Bornstein, David. “Medicine's Search for Meaning.” The New York Times, The New York Times, 18 Sept. 2013, https://opinionator.blogs.nytimes.com/2013/09/18/medicines-search-for-meaning/.

[8] Acerbi A, Lampos V, Garnett P, Bentley RA (2013). The Expression of Emotion in 20th Century Books. PloS ONE.

[9] Bakalis, Nikolaos. Handbook of Greek Philosophy: From Thales to the Stoics: Analysis and Fragments. Fr. 135 De Sensu [On the Senses], 49–83. Victoria: Trafford; 2005.

[10] Cicero MT, Anthon C. Cicero’s Tusculan Disputations. New York: Harper & Brothers; 1879. iv. 6.

[11] Descartes René (1912). A Discourse on Method.

[12] Spinoza BD. Ethic: Demonstrated in Geometrical Order and Divided into Five Parts. London: Humphrey Mulford; 1927. Part V, Appendix V.

[13] Nietzsche F. Beyond Good and Evil. Translated by Kaufmann W. New York: Vintage; 1966. p. 187.

[14] Rorty R. The Linguistic Turn. Chicago: University of Chicago Press; 1967.

[15] Goleman Daniel (1995). Emotional Intelligence.

[16] De Sousa Ronald (1987). The Rationality of Emotion.

[17] Kahneman Daniel (2011). Thinking, Fast and Slow.

[18] Chen D (2007). A Cross-Sectional Measurement of Medical School Empathy. J Gen Intern Med..

[19] Hojat M, Vergare MJ, Maxwell K, Brainard G, Herrine SK, Isenberg GA, Veloski J, Gonnella JS (2009). The Devil is in the Third Year: a longitudinal study of erosion of empathy in medical school. Acad Med.

[20] Artiran Igde, Fusun, and Mustafa Kursat Sahin. “Changes in Empathy during Medical Education: an Example from Turkey.” Pakistan Journal of Medical Sciences, vol. 33, no.5 ,2017, https://www.ncbi.nlm.nih.gov/pmc/articles/PMC5673729/. 10.12669/pjms.335.13074 PMC5673729 29142560

[21] Coulehan J (2005). Viewpoint: Today’s Professionalism: engaging the mind but not the heart. Acad Med.

[22] Underman K, Hirshfield LE (2016). ‘Detached Concern?: Emotional Socialization in Twenty-First Century Medical Education’. Soc Sci Med.

[23] Croskerry P (2022). The Rational Diagnostician and Achieving Diagnostic Excellence. JAMA.

[24] Greenhalgh T (2002). Intuition and evidence—uneasy bedfellows?. Br J Gen Pract.

[25] for a summary of current medical epistemologies - Khushf, G. “A framework for understanding medical epistemologies.” Journal of Medicine and Philosophy, vol. 38, no. 5, 2013, pp. 461–486, 10.1093/jmp/jht044. 10.1093/jmp/jht044 24038643

[26] Guerra-Farfan, Ernesto, et al. “Clinical Practice Guidelines: The Good, the Bad, and the Ugly. Injury, 2022, 10.1016/j.injury.2022.01.047. 10.1016/j.injury.2022.01.047 35135686

[27] Should a Physician Offer Recommendations Based on Experience but Contrary to Current Practice Guidelines? Beth A. Lown, MD and Karen E. Victor, MD, AMA J Ethics. 2018;20(11):E1007–1016. doi: 10.1001/amajethics.2018.1007. 10.1001/amajethics.2018.1007 30499428

[28] Hastings Center Report. The Goals of Medicine: Setting New Priorities. Briarcliff Manor: Hastings Center; 1996;26(6):S1-27. 8970793

[29] Arnetz BB, Goetz CM, Arnetz JE, Sudan S, vanSchagen J, Piersma K, Reyelts F (2020). Enhancing healthcare efficiency to achieve the Quadruple Aim: an exploratory study. BMC Res Notes.

[30] Graham Walker and The NNT Group. “Homepage.” TheNNT, https://www.thennt.com/.

[31] Alex Montero, Audrey Kearney. “Americans' Challenges with Health Care Costs.” KFF, 14 July 2022, https://www.kff.org/health-costs/issue-brief/americans-challenges-with-health-care-costs/.

[32] Judson TJ, Dhruva SS, Redberg RF (2019). Evaluation of technologies approved for supplemental payments in the United States. BMJ.

[33] Shanafelt TD, Hasan O, Dyrbye LN, Sinsky C, Satele D, Sloan J, West CP (2015). Changes in Burnout and Satisfaction With Work-Life Balance in Physicians and the General US Working Population Between 2011 and 2014. Mayo Clin Proc.

[34] LeDoux Joseph E (1996). The Emotional Brain: The Mysterious Underpinnings of Emotional Life.

[35] Evans JSBT, Frankish K. In Two Minds: Dual Processes and Beyond. New York: Oxford University Press; 2009.

[36] Ortony A, et al. The Cognitive Structure of Emotions. Cambridge: Cambridge University Press; 2022.

[37] Brun G, et al. Epistemology and Emotions. London: Routledge; 2016.

[38] Griffiths Paul (1997). What Emotions Really Are: The Problem of Psychological Categories.

[39] Pylyshyn Zenon W (1984). Computation and Cognition.

[40] Damasio Antonio R (1994). Descartes' Error: Emotion, Reason, and the Human Brain.

[41] Clark, John. “The Healing of Philosophy.” 20 Nov. 2020, The Electric Agora, https://theelectricagora.com/2020/11/18/the-healing-of-philosophy/

[42] Ilgen JS, Eva KW, de Bruin A, Cook DA, Regehr G (2019). Comfort with uncertainty: reframing our conceptions of how clinicians navigate complex clinical situations. Adv Health Sci Educ Theory Pract.

[43] as an example of such work, Camras, Linda A, and Amy G Halberstadt. “Emotional Development through the Lens of Affective Social Competence.” Current Opinion in Psychology, vol. 17, 2017, pp. 113–17., 10.1016/j.copsyc.2017.07.003. 10.1016/j.copsyc.2017.07.003 28950956

[44] Shapiro J (2011). Perspective: Does Medical Education Promote Professional Alexithymia? A Call for Attending to the Emotions of Patients and Self in Medical Training. Acad Med.

[45] Foucault Michel (1963). The Birth of the Clinic; an Archaeology of Medical Perception.

[46] Stocker M, Hegeman E (1996). Valuing Emotions.

[47] Djulbegovic, Benjamin, et al. “Dual processing model of medical decision-making.” BMC Medical Informatics and Decision Making, vol. 12, no. 1, 2012, 10.1186/1472-6947-12-94. 10.1186/1472-6947-12-94 PMC3471048 22943520

[48] Hermann, Helena, et al. “Emotion and Value in the Evaluation of Medical Decision-Making Capacity: A Narrative Review of Arguments.” Frontiers in Psychology, vol. 7, 2016, 10.3389/fpsyg.2016.00765. 10.3389/fpsyg.2016.00765 PMC4880567 27303329

[49] Tangney JP, Stuewig J, Mashek DJ (2007). Moral emotions and moral behavior. Annu Rev Psychol.

[50] “Nihilism.” Internet Encyclopedia of Philosophy, https://iep.utm.edu/nihilism/.

[51] Frey JJ (2018). Professional Loneliness and the Loss of the Doctor’s Dining Room. The Annals of Family Medicine.

[52] Jeung DY, Kim C, Chang SJ (2018). Emotional Labor and Burnout: A Review of the Literature. Yonsei Med J.

[53] Berry Diane S, Pennebaker James W (1993). “Nonverbal and Verbal Emotional Expression and Health.”. Psychother Psychosom.

[54] Mangione S, Chakraborti C, Staltari G, Harrison R, Tunkel AR, Liou KT, Cerceo E, Voeller M, Bedwell WL, Fletcher K, Kahn MJ (2018). Medical Students' Exposure to the Humanities Correlates with Positive Personal Qualities and Reduced Burnout: A Multi-Institutional U.S. Survey. J Gen Intern Med.

[55] Moniz Tracy (2021). “The Prism Model for Integrating the Arts and Humanities into Medical Education.”. Acad Med..

[56] Pellegrino ED, Engelhardt HT, Jotterand F (2011). The most humane of the sciences, the most scientific of the humanities. Pellegrino ED: The Philosophy of Medicine Reborn: A Pellegrino Reader.

[57] Panagioti M, Panagopoulou E, Bower P (2017). Controlled Interventions to Reduce Burnout in Physicians: A Systematic Review and Meta-analysis. Key Points Section JAMA Intern Med.

[58] Shem S. The House of God. New York: Berkley Books; 2019. p. 57.

[59] Curlin F, Tollefsen C. The Way of Medicine Ethics and the Healing Profession. Notre Dame: University of Notre Dame Press; 2021.

